# Characteristic and Otopathogenic Analysis of a *Vibrio alginolyticus* Strain Responsible for Chronic Otitis Externa in China

**DOI:** 10.3389/fmicb.2021.750642

**Published:** 2021-12-17

**Authors:** Ke Zhou, Ke-yong Tian, Xin-qin Liu, Wei Liu, Xin-yu Zhang, Jia-yun Liu, Fei Sun

**Affiliations:** ^1^Department of Laboratory Medicine, Institute of Clinical Laboratory Medicine of People’s Liberation Army (PLA), Xijing Hospital, Fourth Military Medical University, Xi’an, China; ^2^Department of Otolaryngology-Head and Neck Surgery, Xijing Hospital, Fourth Military Medical University, Xi’an, China; ^3^Ministry of Education Key Lab of Hazard Assessment and Control in Special Operational Environment and Shaanxi Key Laboratory of Free Radical Biology and Medicine, Department of Occupational and Environmental Health, School of Public Health, Fourth Military Medical University, Xi’an, China

**Keywords:** *Vibrio alginolyticus*, otitis externa, HEI-OC1 cell, tympanic membrane keratinocyte, cochlear organotypic culture, ototoxicity

## Abstract

*Vibrio alginolyticus*, a Gram-negative rod bacterium found in marine environments, is known to cause opportunistic infections in humans, including ear infections, which can be difficult to diagnose. We investigated the microbiological and otopathogenic characteristics of a *V. alginolyticus* strain isolated from an ear exudate specimen obtained from a patient with chronic otitis externa to provide a basis for the future diagnosis of *V. alginolyticus*-associated infections. The identification of *V. alginolyticus* was accomplished using a combination of matrix-assisted laser desorption/ionization-time-of-flight mass spectrometry (MALDI-TOF MS), classical biochemical identification methods, and the use of *Vibrio*-selective media and advanced molecular identification methodologies. Antimicrobial susceptibility testing revealed that the strain was resistant to ampicillin and sensitive to β-lactam, aminoglycosides, fluoroquinolones, and sulfonamide antibiotics. The potential otopathogenic effects of *V. alginolyticus* were determined through the performance of cell viability, cell apoptosis, and cell death assays in tympanic membrane (TM) keratinocytes and HEI-OC1 cells treated with *V. alginolyticus*-conditioned medium using cell-counting kit (CCK)-8 assay, a wound-healing migration assay, Annexin V/propidium iodide (PI) flow cytometric analysis, and terminal deoxynucleotidyl transferase deoxyuridine triphosphate (dUTP) nick-end labeling (TUNEL staining). The results indicated that the identified *V. alginolyticus* strain exerts cytotoxic effects on keratinocytes and HEI-OC1 cells by inhibiting cell proliferation and migration and inducing apoptosis and cell death. To evaluate the ototoxicity of *V. alginolyticus*, the cell density and morphological integrity of hair cells (HCs) and spiral ganglion neurons (SGNs) were analyzed after exposing cochlear organotypic explants to the bacterial supernatant, which revealed the pre-dominant susceptibility and vulnerability of HCs and SGNs in the basal cochlear region to the ototoxic insults exerted by *V. alginolyticus*. Our investigation highlights the challenges associated with the identification and characteristic analysis of the *Vibrio* strain isolated in this case and ultimately aims to increase the understanding and awareness of clinicians and microbiologists for the improved diagnosis of *V. alginolyticus*-associated ear infections and the recognition of its potential otopathogenic and ototoxic effects.

## Introduction

Otitis externa is a common ear affliction characterized by inflammation of the external ear canal, with or without infection (Wipperman, 2014). Infectious acute otitis externa, also referred to as swimmer’s ear, is highly prevalent among young children and swimmers, particularly during the summer and in warm, humid climates. The most common causative pathogens associated with otitis externa are human commensal bacteria, including *Pseudomonas aeruginosa*, *Staphylococcus aureus*, and *Streptococcus pyogenes*, although some rare cases of fungal species have been described, including *Candida* or *Aspergillus*. Otitis media represents a complex, infectious, and inflammatory condition within the middle ear, which is frequently caused by a group of commensal bacteria that colonize the upper respiratory tract, including *Streptococcus pneumoniae*, *Haemophilus influenzae*, and *Moraxella catarrhalis*. Occasionally, infectious agents from the otitis externa, Gram-negative bacteria, and anaerobic bacteria can also cause middle ear infections (Neeff et al., 2016; Szmuilowicz and Young, 2019). Chronic infections of the middle ear result in edema and exudation from the middle ear chamber, the perforation of the tympanic membrane (TM), and the potential disruption of the ossicular chain, resulting in conductive hearing loss (Mittal et al., 2015). As the condition progresses, the generation of bacterial toxins, combined with an excessive immune response (Wong and Ryan, 2015; Jung et al., 2019), can lead to inflammatory damage to the cochlear structures and sensory cells, such as hair cells (HCs) and spiral ganglion neurons (SGNs), ultimately resulting in devastating sensorineural hearing loss (Sun et al., 2020, 2021).

Occasionally, the causative organisms associated with otitis media and otitis externa are airborne or waterborne pathogens transmitted from other infections individuals or environmental sources, such as members of the genera *Mycobacterium* (Nanda et al., 2016; Bhatkar et al., 2017) and *Vibrio* (Kechker et al., 2017). Marine *Vibrio* species, in particular, have been known to cause ear infections associated with exposure to seawater (Pien et al., 1977; Mukherji et al., 2000; Citil et al., 2015). *Vibrio* species, belonging to the Vibrionaceae family, are Gram-negative; straight, curved, or comma-shaped; facultative; anaerobic; motile bacteria that are found ubiquitously in aquatic environments worldwide, including aquaculture conditions, marine coastal waters and sediments, and estuaries. Most *Vibrio* species are non-pathogenic, and only a few cause infectious diseases in aquatic plants, animals, or humans, including 12 species identified as pathogenic to humans, typically causing intestinal and extra-intestinal illnesses. These species include *Vibrio cholera*, *Vibrio parahaemolyticus*, *Vibrio vulnificus*, *Vibrio mimicus*, and *Vibrio alginolyticus* (Baker-Austin et al., 2018). Typically, *V. alginolyticus* is found among coastal flora, in temperate seas and rivers, where they flow into the sea. *V. alginolyticus* is a pathogenic bacterium that affects and contaminates aquatic products in the aquaculture (Liu et al., 2014) and is frequently associated with diarrhea and opportunistic infections in humans, especially during the summer months, including superficial wound infections and eye and ear infections.

A small number of cases describing *V. alginolyticus*-associated ear infections have been documented since 1977 (Pien et al., 1977). However, no cases of *V. alginolyticus*-associated infections have been reported in China or any other Asian country. Due to the rarity of these infections, a limited understanding exists regarding the epidemical tendencies of *V. alginolyticus*-associated ear infections worldwide, with minimal recognition of the biological and pathogenic features. We present a case of chronic otitis externa caused by *V. alginolyticus* in an immunocompetent adult with a history of seawater contact, which represents the first such case reported in China. The microbiological and otopathogenic characteristics of the strain isolated from ear pus specimens obtained from the patient were investigated. Our study highlights the challenges associated with *V. alginolyticus* identification and provides a characteristic analysis of the *Vibrio* strain isolated in this case to ultimately improve the understanding and awareness of clinicians and microbiologists for diagnosing *V. alginolyticus*-associated ear infections and combating its potential otopathogenic effects.

## Case Report

A 42-year-old Chinese man presented to the outpatient clinic of otorhinolaryngology at Xijing Hospital, Fourth Military Medical University, in September 2018, complaining of right ear discharge accompanied by discomfort for 3 years. The patient had a history of seawater contact in August 2015, including underwater diving near the coast of the Yellow Sea near Qingdao City, Shandong Province. After a couple of days, the patient noticed a small volume of clear drainage from both ears, accompanied by mild discomfort and ear pruritus. As the condition progressed, drainage from the left ear gradually vanished within half a month, whereas the drainage from the right side increased and became a thick, purulent exudate containing debris, eventually turning a dark brown or chocolate color. As the ear canal became almost entirely obstructed by the thick excretion, the patient experienced mild hearing loss, coupled with intermittent tinnitus, and experienced a sensation of ear fullness. Without the use of any medications, he removed debris from the right ear using cotton swabs, resulting in a return to normal hearing and the relief of symptoms. Within a few days, new thick drainage would obstruct the ear canal, and the symptoms would reappear. The patient sought health care twice at local hospitals in Xi’an City from Shaanxi Province; however, the underlying illness remained unidentified and did not improve. The discharge from the right ear gradually reduced and turned from brown to white until March 2018, when a low-concentration hydrogen peroxide solution was used to rinse the right ear canal at a local hospital. During the course of the illness, the patient denied any accompanying symptoms, such as fever, chills, headache, vertigo, diarrhea, or flatulence, and he did not have a history of infectious diseases, diabetes, or any immunocompromising condition. Before the diagnosis, the patient underwent audiometric and otoscopic examinations, in addition to radiological examinations using computed tomography imaging to examine the temporal bone. The exudate was collected from his right ear using a sterile swab and was transferred to the clinical microbiology laboratory for examination. All specimen processing and bacteriological analysis procedures were performed with approval from the ethics committee of the Xijing Hospital, Fourth Military Medical University, with assigned number KY20183304-1. The patient provided written informed consent prior to participation in this project.

## Materials and Methods

### Specimen Collection, Processing, and Bacterial Culture

Under otoscopic view, the purulent exudate from the inside of the right ear canal was collected from the patient using a pair of sterile microforceps and obtained using a flocked ESwab transport system (480CE; Copan Diagnostics, Brescia, Italy). Obtained specimens were immediately stored in ESwab Liquid Amies preservation medium and transferred to the clinical microbiology laboratory. The specimen was loaded into the WASPLab laboratory automation system (Copan Diagnostics) for automatic inoculation on 5% sheep blood, chocolate, and MacConkey agar plates (all Autobio, Zhengzhou, China). The inoculated agar plates were moved to a digital imager *via* conveyor belt to obtain the first series of images for the initial plating step. The plates were moved into the WASPLab incubator in an environment with an ambient air temperature of 35°C, where they were maintained for 16 and 40 h. At each incubation time point, a second (16 h) or third series of images (40 h) was acquired and evaluated for bacterial growth by skilled laboratory personnel, according to routine microbiology laboratory protocols.

### Bacterial Identification by Matrix-Assisted Laser Desorption/Ionization-Time-of-Flight Mass Spectrometry

Suspicious pathogen colonies were identified by matrix-assisted laser desorption ionization-time of flight mass spectrometry (MALDI-TOF MS). Briefly, a portion of a single bacterial colony was applied directly to a target slide (bioMérieux, Marcy l’Etoile, France) using a disposable 1-ml loop. Immediately, 1 ml of Vitek MS-CHCA matrix solution (α-cyano-4-hydroxycinnamic acid; bioMérieux) was added directly to the bacterial film and allowed to dry at room temperature, followed by mass spectrometric analysis. The mass spectrometer was calibrated using a freshly prepared *Escherichia coli* ATCC 8739 reference strain. Individual colonies were analyzed by the Vitek MS system (bioMérieux) in linear positive-ion mode with Vitek MS Acquisition Station software (version 2.0; bioMérieux). The similarities between each sample and the reference isolates in the MS-ID database were calculated and expressed as an optimized quantitative value (confidence value).

### Bacterial Identification by Chromogenic Media and Biochemical Characteristics

For confirmation of the bacterial identification obtained from MALDI-TOF MS analysis, the presumptive *Vibrio* strain was sub-cultured on CHROMagar™ Vibrio agar (CHROMagar, Paris, France). Concurrently, clinical strains of *V. vulnificus* and *V. cholera* were inoculated on chromogenic agar plates to serve as controls. All plates were incubated at 35°C and were observed to determine colony morphologies and coloration at 24 and 48 h, in accordance with the instructions of the manufacturer.

The confirmatory identification of isolates was also performed using two commercially available biochemical identification systems, including API 20E and 20NE strips and the Vitek-2 Compact system (bioMérieux). According to the recommendations of the manufacturer, a single bacterial colony was resuspended in 0.85% sodium chloride (NaCl; bioMérieux) using sterile swabs to prepare a 0.5-McFarland suspension. The suspension was inoculated into the eight left-sided microtubes on an API 20NE strip and all of the microtubes on an API 20E strip. Subsequently, 200 μl of suspension was pipetted into 7-ml API AUX medium (bioMérieux) and inoculated into the remaining 12 right-sided microtubes on the API 20NE strip. Supplementary investigations of cytochrome oxidase were performed using Microbact Oxidase strips (Oxoid, Basingstoke, United Kingdom). All inoculated API strips were incubated at 35°C for 24 h, and the species identification results were obtained using the API database included in APILAB Plus software (bioMérieux). Simultaneously, the 0.5-McFarland bacterial suspension was transferred onto a Vitek-2 Gram-negative identification card (GN; bioMérieux), and the card was loaded into the Vitek-2 Compact system for bacterial identification. The *E. coli* ATCC 25922 strain was used as quality control.

### 16S rRNA Gene Sequencing and Species Identification

Further confirmation of bacterial identification was performed through the polymerase chain reaction (PCR)-based amplification of the 16S rRNA gene using the universal primers 27F (5′-AGAGTTTGATCCTGGCTCAG-3′) and 1492R (5′-TACGGCTACCTTGTTACGACTT-3′). PCR conditions consisted of 95°C for 5 min; 35 cycles of 95°C for 1 min and 55°C for 1 min; annealing at 72°C for 2 min; and a final extension step at 72°C for 10 min. PCR amplification was performed in an ABI 7500 thermocycler (Applied Biosystems, Foster City, CA, United States), and the resulting amplified product was verified by size separation on a 1% agarose gel. The DNA sequences of the purified PCR products were identified using an ABI Prism 3700 Genetic Analyzer (Applied Biosystems) and compared with the sequences deposited in the GenBank database from the National Center for Biotechnology Information (NCBI) by using BLAST.^1^

### Antimicrobial Susceptibility Testing

Following identification, the *Vibrio* isolate was subjected to antimicrobial susceptibility testing to analyze its antibiotic-resistant phenotype. According to the recommendations of the manufacturer, 145 μl of freshly prepared 0.5-McFarland bacterial suspension was diluted into 3 ml of 0.85% NaCl and transferred onto a Vitek-2 Gram-negative antimicrobial susceptibility testing card (AST-GN13; bioMérieux). Subsequently, the card was loaded into the Vitek-2 Compact system for automatic incubation, and the minimum inhibitory concentration (MIC; μg/ml) results were reported through the software. Antimicrobial susceptibility was interpreted according to the current M45-S3 guidelines of the Clinical and Laboratory Standards Institute (CLSI). The *E. coli* ATCC 25922 strain was used as quality control for antimicrobial susceptibility testing using the broth microdilution method.

### Preparation of Bacterial Supernatant

For the generation of bacterial supernatant, the clinical *Vibrio* strain was cultured in brain-heart infusion broth (BHI; BD Laboratories, Franklin Lakes, NJ, United States) supplemented with 0.85% NaCl at 35°C for 16 h. Subsequently, the culture broth was diluted with fresh BHI medium at a ratio of 1:10, allowing bacteria to grow for 5 h at 35°C, to reach the logarithmic growth phase. The bacterial culture was pelleted by centrifugation at 5,000 × *g* and 4°C for 10 min, and 1 × 10^6^ colony-forming units (CFU)/ml bacteria were resuspended in high-glucose Dulbecco’s modified Eagle’s medium (DMEM) supplemented with 10% fetal bovine serum (FBS; both Thermo Fisher Scientific, MA, United States) or Basal Medium Eagle (Sigma-Aldrich/Merck KGaA, Darmstadt, Germany). After incubating for 8 h, the bacterial suspension was centrifuged, and the resulting supernatant was filtered through a 0.22-μm filter (Millex GP; Millipore/Merck KGaA, Darmstadt, Germany). The accuracy of the bacterial concentration was confirmed by quantitative cultures on 5% sheep blood agar plates.

### Animal Care and Tissue Preparation

Post-natal day 3 (P3) Sprague–Dawley rats were obtained from the Laboratory Animal Center of the Fourth Military Medical University. The experimental procedures for the animal study were approved by the Animal Care Committee of Fourth Military Medical University. To minimize the number of animals used and their suffering, the rat pups were sacrificed by rapid decapitation, and their skulls were opened midsagitally. For the preparation of TM keratinocyte cultures, both sides of the external ears were removed, and the tympanic bulla was isolated under a dissecting microscope (Stemi 508; Carl Zeiss, Göttingen, Germany). The TM was dissected and rinsed with ice-cold Hank’s Balanced Salt Solution (HBSS) containing 2% penicillin–streptomycin solution (both Thermo Fisher Scientific, MA, United States) prior to processing. For the preparation of cochlear organotypic cultures, rat cochleae were promptly separated from the temporal bone, rinsed with ice-cold HBSS, and collected for further use.

### Tympanic Membrane Keratinocyte and House Ear Institute-Organ of Corti 1 Cell Cultures and Bacterial Supernatant Treatment

Harvested TMs were adhered to a single 35-mm culture dish with a glass-bottom (15-mm glass diameter; NEST Biotechnology, Wuxi, China) previously coated with Cell-Tak (Corning Life Science, Corning, NY, United States), with the lateral surface of the TM facing down. Defined keratinocyte-serum-free medium (dKSFM; Thermo Fisher Scientific) containing antibiotics was added to the TM explant cultures, and the cultures were incubated in an atmosphere of 5% CO_2_ and 95% humidity at 35°C. Keratinocyte outgrowths from each explant were allowed to form a homogeneous cell monolayer. Upon reaching 80–90% confluence, cells were passaged by enzymatic digestion and fed with high-glucose DMEM containing 10% FBS. House Ear Institute-Organ of Corti 1 (HEI-OC1) cells (a gift from Dr. Renjie Chai, Southeast University, Nanjing, China) were cultured in high-glucose DMEM containing 10% FBS in an atmosphere of 5% CO_2_ and 95% humidity at 33°C.

Tympanic membrane keratinocytes and HEI-OC1 cells within passages 3–5 were used for further experiments. Cells from experimental cultures were incubated with fresh medium supplemented with bacterial supernatant at a 1:1 ratio and maintained for 24 h (keratinocytes) or 12 h (HEI-OC1 cells) prior to further analysis. Cells incubated in supernatant-free medium alone were used as the baseline control.

### Cell Viability Assay

The viabilities of TM keratinocytes and HEI-OC1 cells were quantitatively assessed using the cell-counting kit-8 (CCK-8; Beyotime, Beijing, China). Both types of cells were seeded in 96-well plates at a density of 5.0 × 10^3^ cells/well using three replicates per group and subjected to the previously described treatments. After cultivation, the cell culture was incubated with 100 μl of fresh medium containing 10 μl CCK-8 reagent in each well for 60 min at 37°C. Cell viability was calculated by measuring the optical densities of each well at 450 nm using a microplate reader (Bio-Rad Laboratories, Hercules, CA, United States).

### Wound-Healing Migration Assay

Tympanic membrane keratinocytes were seeded into the reservoirs of a two-well culture insert that was adhered to a 35-mm glass-bottom dish (Ibidi, Martinsried, Germany), at a density of 5.0 × 10^3^ cells/insert, and cultured overnight at 37°C. Once the cells reached confluence, the insert was removed from each dish using a pair of forceps, and the dish was gently rinsed with phosphate-buffered saline (PBS) to remove cell debris. The cells were then cultured in fresh medium, either with or without bacterial supernatant supplementation, and placed into a Cytation 5 Cell Imaging Multi-Mode Reader (BioTek Instruments, Winooski, VT, United States). Phase-contrast images of the wound gaps were viewed and captured under a ×4 objective lens using Gen5 software (BioTek Instruments) at 1-h intervals over 24 h. The wound gap areas at times 0 and 24 h were analyzed with ImageJ software (version 1.46r; National Institutes of Health, Bethesda, MD, United States) to calculate repair rates (expressed in μm^2^/h).

### Cell Death Analysis by Flow Cytometry

Apoptosis rates for TM keratinocytes and HEI-OC1 cells treated with bacterial supernatant were detected by flow cytometry according to the protocol supplied with the FITC Annexin V/propidium iodide (PI) apoptosis detection kit (BD Pharmingen, San Diego, CA, United States). In brief, cells were plated in a 60-mm culture dish at a density of 1.0 × 10^6^ cells/dish and incubated in medium with or without bacterial supernatant supplementation at 37°C in a 5% CO_2_ atmosphere. Cells from each experimental group were dissociated with 0.25% trypsin, pelleted by centrifugation, and washed twice with ice-cold PBS. For flow cytometric analysis, harvested cells were resuspended in 1× binding buffer at a concentration of 1 × 10^6^ cells/ml. A 100-μl volume of binding buffer containing 5 μl of Annexin V-FITC and 5 μl of PI solution was added to each sample and incubated for 15 min in the dark. After adding an additional 400 μl of binding buffer, all samples were evaluated using a flow cytometer (FACS Canto™ II; BD Biosciences).

### Cell Apoptosis Analysis by Terminal Deoxynucleotidyl Transferase-Mediated Deoxyuridine Triphosphate Nick-End Labeling Assay

For immunofluorescent analysis of cell apoptosis, TM keratinocytes and HEI-OC1 cells were plated in a 35-mm glass-bottom culture dish (NEST Biotechnology) at a density of 5.0 × 10^4^ cells/dish and treated with bacterial supernatant, as described previously. Apoptosis was determined by terminal deoxynucleotidyl transferase-mediated dUTP nick-end labeling (TUNEL) using an *in situ* cell death detection kit (TMR red; Roche, Nutlet, NJ, United States), according to the instructions of the manufacturer. Briefly, cells in the culture dishes were fixed in 4% paraformaldehyde for 20 min at room temperature, and staining was performed using a TUNEL reaction mixture at 37°C for 60 min in a humidified atmosphere. After washing, the cell samples were permeabilized and blocked with a PBS solution containing 5% bovine serum albumin (BSA; Sigma-Aldrich) and 0.1% Triton X-100. Cells were then labeled at 4°C overnight using antibody solution (1% BSA and 0.1% Triton X-100 in PBS) containing the following primary antibodies: monoclonal mouse anti-cytokeratin (CK)-19 primary antibody (diluted 1:200; GeneTex, Irvine, CA, United States) for keratinocytes, or polyclonal rabbit anti-Myosin-VIIa antibody (diluted 1:1,000; Proteus Biosciences, Ramona, CA, United States) for HEI-OC1 cells. Immunolabeling was further visualized with appropriate secondary antibodies: Alexa Fluor 488-conjugated donkey anti-mouse IgG or Alexa Fluor 488-conjugated donkey anti-rabbit IgG (both diluted 1:500; Thermo Fisher Scientific) diluted in antibody solution for 2 h at room temperature. In parallel, all specimens were randomly processed without incubation with primary antibodies, which served as the negative control. After nuclear counterstaining with Hoechst 33342 (diluted 1:1,000; Thermo Fisher Scientific) for 15 min, all samples in the culture dishes were mounted with Prolong Gold anti-fade mounting medium (Thermo Fisher Scientific) and randomly visualized on a confocal microscope system (FV1000; Olympus, Tokyo, Japan). The confocal images were converted to TIFF format using FV10-ASW software (version 4.2; Olympus) and processed for optimal brightness and contrast with Adobe Photoshop software (CC 2020; Adobe Systems, San Jose, CA, United States). The TUNEL/Hoechst double-positive cells were calculated, and the number was normalized against the total number of viable cells to determine the TUNEL-positive rate.

### Cochlear Organotypic Culture and Bacterial Supernatant Treatment

The experimental protocol for cochlear organotypic cultures was performed as previously described (Ding et al., 2011; Prakash Krishnan Muthaiah et al., 2017). A drop (20 μl) of cool, type I rat tail collagen gel (Corning Life Science) in 10× basal medium Eagle (BME; Sigma-Aldrich) supplemented with 2% sodium carbonate (Sigma-Aldrich) at a 9:1:1 ratio was placed in a 35-mm culture dish at room temperature for 15 min to initiate gelation. Subsequently, 1.2 ml of serum-free medium, consisting of 1× BME (Sigma-Aldrich), 1% serum-free supplement (100× insulin-transferrin-selenium-X supplement; Sigma-Aldrich), 1% BSA, 2 mM glutamine, 5 mg/ml of glucose, and 1% antibiotics, was added to cover the collagen gel in each dish. Using a dissecting microscope, the membranous cochlea was revealed from the cochlear capsule of a P3 rat. After removing the spiral ligament and stria vascularis, the remaining auditory sensory epithelium containing the organ of Corti and SGNs was placed evenly on the collagen gel and maintained overnight at 37°C with 5% CO_2_ before exposure to the bacterial supernatant. On the following day, the cochlear explants were incubated with fresh medium supplemented with or without bacterial supernatant at a 1:1 ratio and maintained for 4 h prior to immunofluorescent analysis. After fixation and permeabilization, HCs and SGNs from the organotypic cultures were labeled with monoclonal mouse anti-β-III Tubulin (TUJ1) antibody (diluted 1:500; GeneTex) and polyclonal rabbit anti-Myosin-VIIa antibody (diluted 1:1,000), followed by incubation with Alexa Fluor 647-conjugated donkey anti-mouse IgG and Alexa Fluor 488-conjugated donkey anti-rabbit IgG (both diluted 1:500; Thermo Fisher Scientific). In parallel, cochlear specimens were randomly processed without incubation with primary antibodies, which served as a negative control. Alexa Fluor 594-conjugated phalloidin (diluted 1:500; Thermo Fisher Scientific) was used for F-actin labeling within 15 min. After counterstaining with Hoechst 33342, all explant samples were subsequently mounted with Prolong Gold medium and photographed under a confocal microscope.

### Statistical Analysis

All experimental data are shown as the mean ± standard deviation (SD) and were analyzed using the Statistical Program for Social Science software (SPSS, version 26.0; IBM Inc., Armonk, NY, United States). Two-tailed, unpaired Student’s *t*-tests were used to assess significant differences between two groups. Values of *p* less than 0.05 (*p* < 0.05) were considered significant.

## Results

### Clinical Diagnosis, Treatment, and Outcome of the Patient

**FIGURE 1:**
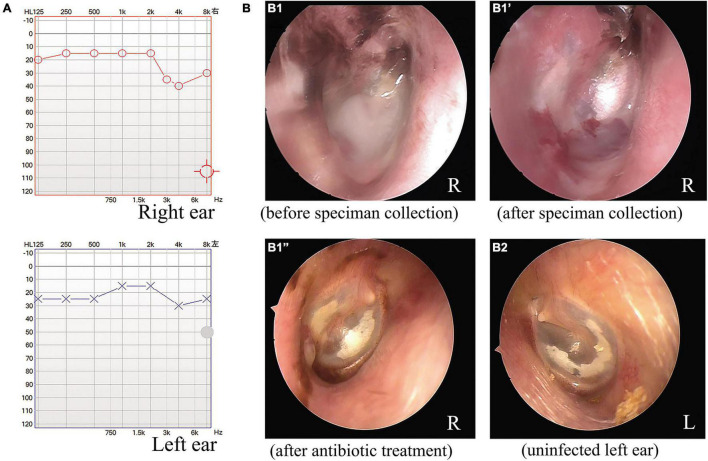
Audiometric and otoscopic examination of a patient with a chronic ear infection. The audiometric evaluation indicated mild hearing impairment associated with the chronic ear infection of the patient **(A)**. Otoscopic examination of the right ear of the patient before **(B1)** and after microbiological specimen collection **(B1′)** and after antimicrobial treatment **(B1^”^)**. Otoscopic view of the uninfected left ear of the patient after antimicrobial treatment for the right ear **(B2)**.

As depicted in Figure 1A, the audiometric evaluation of the patient displayed a mild sensorineural hearing loss, with a mild drop at high frequencies in both ears. Computed tomography of the temporal bone showed normal middle ear and mastoid air cells, with no signs of cholesteatoma or otomastoiditis. An otoscopic examination revealed some white purulent discharge on the deep wall of the external auditory canal and the lateral surface of the TM in the right ear (Figure 1B1). The exudate was removed with a pair of microforceps, collected using a sterile swab, and transferred to the clinical microbiology laboratory for microorganism culture. After sampling, otoscopic visualization of the right ear showed an intact, retracted, and opaque TM, with local calcification in the pars tensa and some granulated tissue on the deep posterior wall of the ear canal near the TM (Figure 1B1′). The patient was prescribed topical ciprofloxacin eardrops, applied three times per day. After 4 days, the organism recovered from the exudate sample was identified as *V. alginolyticus*, and the strain showed susceptibility to most antimicrobial agents, including levofloxacin. The patient was finally diagnosed with chronic otitis externa caused by *V. alginolyticus*. Following a successful course of antibiotics, the patient eventually recovered after a follow-up of 1 week. The otoscopic examination also demonstrated local calcification in the intact and thickened TMs on both sides of the ears (Figures 1B1″,B2), and an additional ear swab culture was negative for *V. alginolyticus*.

### Laboratory Identification and Microbiological Characteristics of *Vibrio alginolyticus*

**FIGURE 2:**
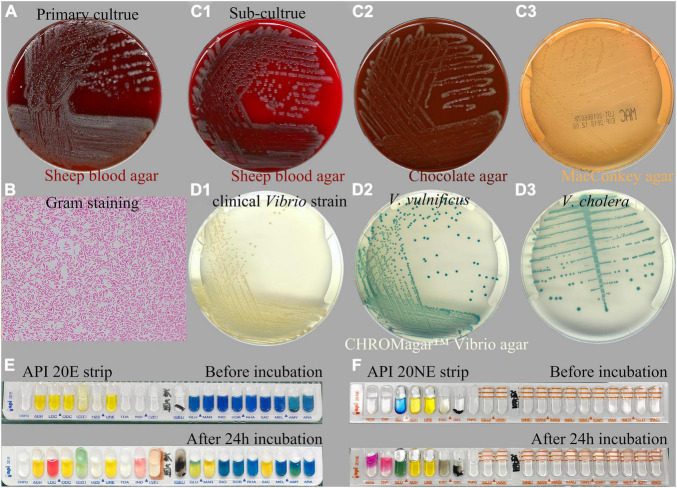
Microbiological characteristics of the *Vibrio alginolyticus* strain responsible for chronic ear infection. Cultures of the specimen obtained from the ear infection drainage revealed the large growth of a Gram-negative bacterium **(A)**. Gram staining of cultured *Vibrio* colonies showed a Gram-negative, curved, and rod-shaped morphology **(B)**. Sub-culture of *Vibrio* on sheep blood **(C1)**, chocolate **(C2)**, and MacConkey agar media **(C3)**. For confirmation, the isolate appeared to be creamy, as expected for *V. alginolyticus* when sub-cultured on a CHROMagar™ Vibrio-selective agar **(D1)**, whereas the control isolates of *Vibrio vulnificus* **(D2)**, and *Vibrio cholera* **(D3)** appeared to be green-blue to turquoise-blue. Biochemical investigation of the tested strain using API 20E **(E)** and 20NE **(F)** strips identified the isolate as *V. alginolyticus*.

Two days after plating, cultures of the exudate specimen obtained from the infected ear and inoculated on sheep blood agar revealed a large growth of pure, gray, non-hemolytic bacterium colonies and some scattered white Staphylococcus colonies (Figure 2A). Gram staining of the isolate showed a Gram-negative, curved, rod-shaped morphology resembling *Vibrio* spp. (Figure 2B). The strain was identified at the species level using MALDI-TOF MS analysis, revealing a 99.9% coefficient of variation for identification as *V. alginolyticus* (Supplementary Figure 1). Sub-cultured strains were identified as grayish colonies on sheep blood (Figure 2C1) and chocolate (Figure 2C2) agar media and as transparent, glucose-non-fermenting colonies on MacConkey agar plate (Figure 2C3). For confirmation of the *V. alginolyticus* identification, the isolate was sub-cultured overnight on a CHROMagar™ Vibrio agar plate. The colonies that grew on the chromogenic media appeared creamy or colorless, as expected for *V. alginolyticus* (Figure 2D1), whereas the control colonies of *V. vulnificus* (Figure 2D2) and *V. cholera* (Figure 2D3) appeared green-blue to turquoise-blue.

Additionally, a biochemical investigation of the identified *Vibrio* strain was performed using a Vitek-2 GN ID card on a Vitek-2 Compact system, and the API 20E (Figure 2E) and 20NE (Figure 2F) system also identified the isolate as *V. alginolyticus*, which was positive for the following tests: growth in nutrient broth supplemented with 1 and 6% NaCl; production of acid from glucose, mannitol, and saccharose; utilization of glucose, mannitol, N-acetyl-glucosamine, gluconate, and malate; lysine decarboxylase; cytochrome oxidase; nitrate reduction; gelatin hydrolysis; indole; and motility. By contrast, the strain was negative for growth in nutrient broth without NaCl supplementation; H_2_S production; production of acid from inositol, sorbitol, rhamnose, melibiose, amygdalin, arabinose, cellobiose, lactose, and salicin; utilization of arabinose, mannose, maltose, caprate, adipate, citrate, and phenylacetate; urea hydrolysis (urease); arginine dihydrolase; ornithine decarboxylase; Voges–Proskauer (VP); β-galactosidase (4-nitrophenyl-β-D-glucopyranoside, PNPG); and O-nitrophenyl-b-D-galactopyranoside (ONPG). The isolate was biochemically identical to the reactions reported for *V. alginolyticus* among four common pathogenic *Vibrio* species listed in Table 1. Molecular identification using 16S rRNA gene sequencing was also performed, indicating that the species was most closely related to *V. alginolyticus* (Supplementary Data), and the overall identity score was 100%, with no mismatches.

**TABLE 1:** Comparison of biochemical responses between the clinical *Vibrio* isolate and four common pathogenic *Vibrio* species^a^.

Test	Positive rate for strains (%)^b^				
	Tested *Vibrio* isolate	*V. alginolyticus*	*V. cholera*	*V. vulnificus* ^d^	*V. parahaemolyticus*
Indole (HIB)^c^	+	85	99	97	98
VP^c^	−	95	75	0	0
Moeller’s arginine^c^	−	0	0	0	0
Moeller’s lysine^c^	+	99	99	99	100
Moeller’s ornithine^c^	−	50	99	55	95
Motility	+	99	99	99	99
Gelatin hydrolysis (22°C)^c^	+	90	90	75	95
D-glucose, gas production	−	0	0	0	0
**Acid production from:**					
L-arabinose	−	1	0	0	80
Cellobiose	−	3	8	99	5
Lactose	−	0	7	85	1
Myo-inositol	−	0	0	0	0
Salicin	−	4	1	95	1
Sucrose	+	99	100	15	1
ONPG	−	0	94	75	5
**Salt tolerance (growth in nutrient broth with):**					
0% NaCl	−	0	100	0	0
6% NaCl	+	100	53	65	99
O/129 susceptibility^e^	−	19	99	98	20

*^a^Main biochemical responses of four Vibrio species were modified from the following book: James H. Jorgensen, Michael A. Pfaller, editors: Manual of Clinical Microbiology, 11th Edition. ASM Press, 2015, p. 764.*

*^b^Percentage of strains positive after 48 h of incubation at 36°C unless otherwise indicated. Most positive reactions occur within 24 h.*

*^c^Sodium chloride, 1%, is added to specific tests.*

*^d^Biogroup 1 strains.*

*^e^Zone of inhibition present (disk content, 150 μg).*

*HIB, heart infusion broth; VP, Voges–Proskauer; ONPG, o-nitrophenyl-b-D-galactopyranoside; NaCl, sodium chloride.*

Antimicrobial susceptibility testing of the *V. alginolyticus* strain was performed using a Vitek-2 AST-GN13 card on a Vitek-2 Compact system and interpreted according to the CLSI M45-S3 breakpoints for *Vibrio* spp. The results revealed that the isolate was sensitive to piperacillin, ampicillin/sulbactam, piperacillin/tazobactam, ceftazidime, cefepime, imipenem, meropenem, gentamicin, amikacin, levofloxacin, ciprofloxacin, and trimethoprim–sulfamethoxazole but resistant to ampicillin (Table 2).

**TABLE 2:** Antimicrobial susceptibility results of the *Vibrio alginolyticus* strain.

Antimicrobial	MIC (μg/ml)	Susceptibility
Ampicillin	≥32	R
Piperacillin	≤4	S
Ampicillin/sulbactam	8	S
Piperacillin/tazobactam	≤4	S
Ceftazidime	≤1	S
Cefepime	≤1	S
Imipenem	≤1	S
Meropenem	≤0.25	S
Gentamicin	≤1	S
Amikacin	≤2	S
Levofloxacin	≤0.25	S
Ciprofloxacin	≤0.25	S
Trimethoprim-sulfamethoxazole	≤20	S

*MIC, minimum inhibitory concentration; R, resistant; S, susceptible.*

### Cytotoxic Effects Induced by *Vibrio alginolyticus* in Tympanic Membrane Keratinocytes and House Ear Institute-Organ of Corti 1 Cells

**FIGURE 3:**
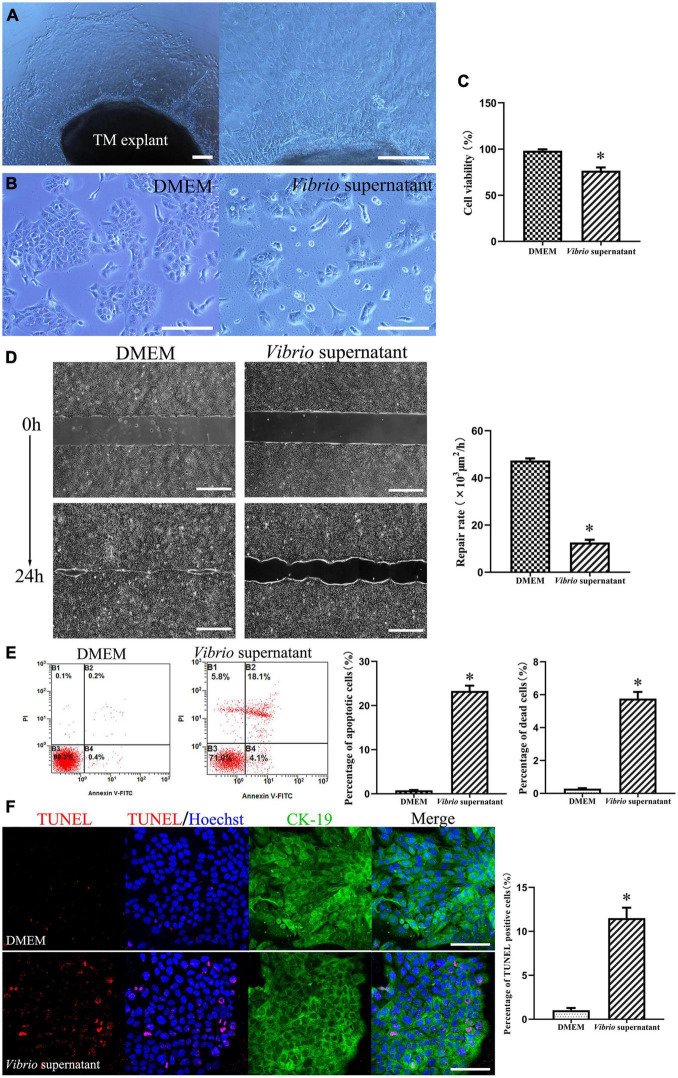
Cytotoxic effects on tympanic membrane (TM) keratinocytes induced by *Vibrio alginolyticus*. The keratinocyte outgrowth from the TM explant formed a monolayer of epithelial cells, displaying a cobblestone-like morphology **(A)**. Cytopathic effects in keratinocytes after exposure to the *V. alginolyticus* culture supernatant for 24 h **(B)**. Quantitative analysis of cell viability measured by cell-count kit-8 (CCK-8) assay after exposure to bacterial supernatant **(C)**. Wound-healing migration assay was performed by analyzing repair rates of wound gaps **(D)**. To detect cell death and apoptosis, the proportion of apoptotic and dead cells was counted by flow cytometric analysis with Annexin V/propidium iodide (PI) double staining **(E)** and the number of terminal deoxynucleotidyl transferase-mediated dUTP nick-end labeling (TUNEL)-positive stained apoptotic cells was calculated **(F)**. The results are expressed as the mean ± standard deviation (SD) [*p < 0.05, vs. control/Dulbecco’s modified Eagle’s medium (DMEM)]. Scale bars: A, B = 200 μm, *D* = 500 μm, *F* = 100 μm.

To explore the potential cytotoxic effects of *V. alginolyticus* on rat TM keratinocytes, we isolated keratinocytes from TM tissue explant cultures obtained from P3 rats, and the cells were treated with *V. alginolyticus* culture supernatants diluted 1:1 for 24 h. The keratinocyte outgrowth from the TM explant formed a monolayer of epithelial cells that displayed a tightly packed, cobblestone-like morphology (Figure 3A). When maintained in normal medium, the passaged keratinocytes proliferated promptly and formed large numbers of homogenous clones. After exposure to the bacterial supernatant for 24 h, the proliferation of keratinocytes was markedly compromised, accompanied by a cytopathic effect that manifested as the shrinkage and rounding of individual cells (Figure 3B). To quantify cell proliferation, CCK-8 assays were performed, which revealed that the cell viability of keratinocytes treated with *Vibrio* supernatant decreased compared with that of the control culture (Figure 3C). To assess the functionality of TM keratinocytes, a time-lapse wound-healing migration assay was performed, in which the repair rates of wound gaps were analyzed. As shown in Figure 3D, healthy epidermal cells maintained under control conditions proliferated and migrated to close the wound gap created by the two-well culture insert over 24 h (Supplementary Video 1). Conversely, the wound-healing repair rate for keratinocytes cultured in *Vibrio*-derived conditioned medium was significantly reduced relative to the normal control (Supplementary Video 2). To investigate the cell death mechanisms induced by *Vibrio* supernatant, keratinocytes incubated under different conditions were stained with Annexin V-FITC/PI or TUNEL. Flow cytometric analysis was performed with Annexin V/PI double staining, which indicated that the proportions of apoptotic and dead cells were significantly increased in the bacterial supernatant-treated culture compared with the control culture (Figure 3E). In addition, the number of TUNEL-positive apoptotic cells was significantly higher after exposure to *Vibrio* supernatant compared with control conditions (Figure 3F).

**FIGURE 4:**
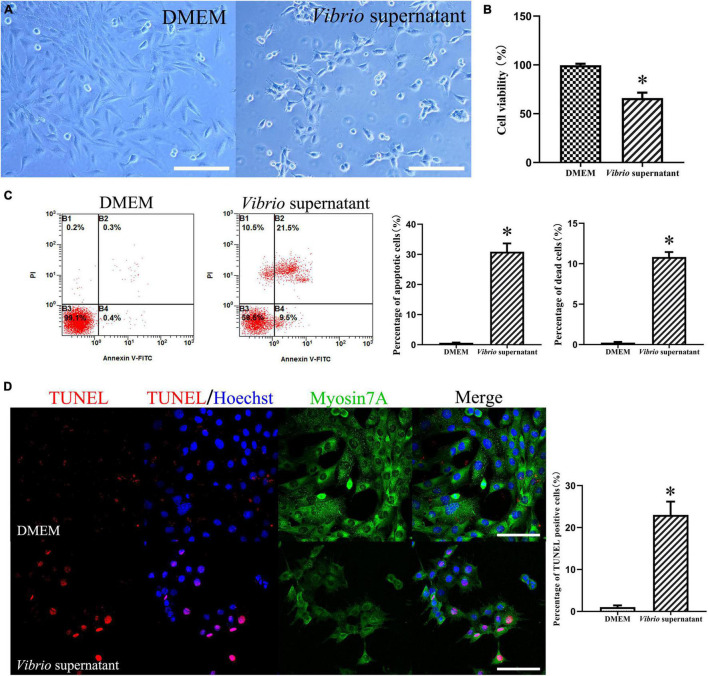
Cytotoxic effects on House Ear Institute-Organ of Corti 1 (HEI-OC1) cells induced by *Vibrio alginolyticus*. Cytopathic effects of HEI-OC1 cells treated with *Vibrio* supernatant for 12 h **(A)**. Quantitative analysis of cell viability measured by cell-counting kit (CCK)-8 assay after exposure to bacterial supernatant **(B)**. The proportion of apoptotic and dead cells was counted by flow cytometric analysis with Annexin V/propidium iodide (PI) double staining **(C)**, and the number of TUNEL-positive stained apoptotic cells was calculated **(D)**. The results are expressed as the mean ± standard deviation (SD) [*p < 0.05, vs. control/Dulbecco’s modified Eagle’s medium (DMEM)]. Scale bars: *A* = 200 μm, *D* = 100 μm.

To evaluate the possible effects of *V. alginolyticus* on HEI-OC1 cells, we treated the cells with the bacterial supernatant at the same concentration for 12 h. After exposure to *Vibrio-*derived conditioned medium, HEI-OC1 cells appeared as grape-like aggregates of swollen, refractile cells (Figure 4A). The results from the CCK-8 assay demonstrated significantly decreased viability among HEI-OC1 cells treated with supernatant (Figure 4B). Similarly, exposure to bacterial supernatant resulted in a significantly higher ratio of apoptotic and dead HEI-OC1 cells compared with the control condition (Figure 4C). In addition, *Vibrio* supernatant treatment increased the number of dead cells that exhibited TUNEL/Hoechst double-positive staining and condensed nuclei (Figure 4D). These results indicated that the *V. alginolyticus* strain exerted cytotoxic effects against TM keratinocytes and HEI-OC1 cells by inhibiting cell proliferation and migration and inducing cell apoptosis and death.

### Ototoxic Effects on Cochlear Organotypic Cultures Induced by *Vibrio alginolyticus*

**FIGURE 5:**
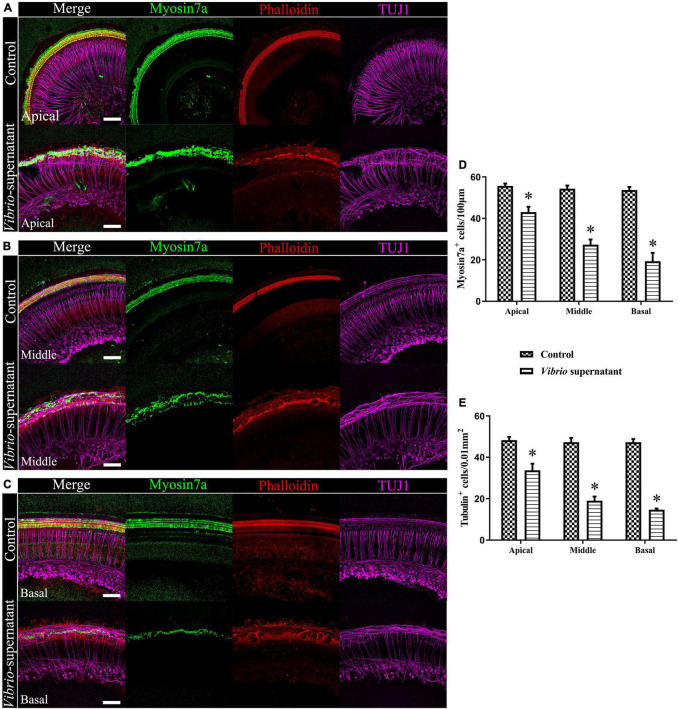
Ototoxic effects on cochlear organotypic explant cultures induced by *Vibrio alginolyticus*. Ototoxic damage was observed at the apical **(A)**, middle **(B)**, and basal **(C)** turns of the cochlear explants. The numbers of Myosin 7a^+^, phalloidin^+^ hair cells (HCs) **(D)**, and β-III Tubulin (TUJ1)^+^ spiral ganglion neurons (SGNs) were quantified **(E)**. The results are expressed as the mean ± standard deviation (SD) (*p < 0.05, vs. control). Scale bars = 100 μm.

To study the ototoxic effects of *V. alginolyticus* on sensory HCs and SGNs, we analyzed the cell density and morphological integrity of cochlear organotypic explants after exposure to a 1:1 dilution of bacterial culture supernatant for 4 h. As shown in Figure 5, the ototoxic damage was quantified (Figures 5D,E) at the apical (Figure 5A), middle (Figure 5B), and basal (Figure 5C) turns of the cochlear explants, and the effects on HCs and SGNs were assessed by immunostaining the whole mounts with the HC marker Myosin 7a, a phalloidin dye that stains stereocilia bundles, and the neuronal marker TUJ1. Significantly reduced HC and SGN survival was observed in the basal turn compared with the corresponding cochlear regions in control cultures. In detail, the morphology of Myosin 7a-labeled inner HCs and phalloidin-labeled stereocilia bundles were entirely absent, the density of TUJ1-labeled auditory neurons and nerve fibers were significantly reduced, and the outer spiral fiber bundles of type II SGNs were swollen and disarranged (Figure 5C). By contrast, most of the HCs and SGNs were maintained in the apical turns of the cochlear explants exposed to the bacterial supernatant (Figure 5A). These results indicated a high susceptibility of HCs and SGNs to *V. alginolyticus*-induced ototoxicity *in vitro*, with increased vulnerability in the basal and middle cochlear regions.

## Discussion

Similar to other halophilic marine *Vibrio* species, including *V. vulnificus* and *V. parahaemolyticus*, *V. alginolyticus* has been identified as pathogenic to humans and animals, causing serious seafood-poisoning or even fatal extra-intestinal infections, such as necrotizing soft-tissue infections, bacteremia, septic shock, and multiple organ failures (Baker-Austin et al., 2018). Acute gastroenteritis with diarrhea was the most common clinical presentation of *V. alginolyticus* infections associated with the ingestion of raw seafood (Uh et al., 2001). Recently, the reported incidence of extra-intestinal infections due to *V. alginolyticus* through exposure to seawater has dramatically increased, including skin wound infections (Bultmann et al., 2016), conjunctivitis (Lessner et al., 1985), endophthalmitis (Li et al., 2009), peritonitis (Taylor et al., 1981), necrotizing fasciitis (Gomez et al., 2003), osteomyelitis (Barbarossa et al., 2002), intracranial infection (Opal and Saxon, 1986), bacteremia (Ruiz and Agraharkar, 2003), and ear infections. These infections increase significantly during the summer due to the increased temperature of seawater. In addition, climate change over recent years has increased the abundance of these bacteria in both tropical and temperate waters.

Ear infections (Levine et al., 1993), including otitis externa (García-Martos et al., 1993; Orden and Franco, 1993; Mukherji et al., 2000; Schets et al., 2006), myringitis (Citil et al., 2015), and otitis media (Ciufecu et al., 1979; Hasyn et al., 1987; Tsakris et al., 1995; Ardiç and Ozyurt, 2004; Feingold and Kumar, 2004), caused by *V. alginolyticus* have increased in prevalence in subtropical regions. All reported cases, as summarized in Table 3, occurred in countries in the northern hemisphere rather than those in the southern hemisphere, with the majority of cases occurring in countries with warm seacoasts, such as the Mediterranean countries. *V. alginolyticus*-associated ear infections have a similar clinical presentation as other pyogenic pathogens, such as *P. aeruginosa*, *S. aureus*, *Proteus mirabilis*, and *E. coli*, which were the most commonly isolated bacteria collected from patients with suppurative otitis. In addition, a few cases of ear infections caused by other *Vibrio* spp., such as *V. cholerae* (non-O1/O139 strains) (Kechker et al., 2017), *V. fluvialis* (Chen et al., 2012), *V. mimicus* (Baker-Austin et al., 2018), and *V. vulnificus* (Sato et al., 2001), have also been documented.

**TABLE 3:** Case reports of ear infections caused by *Vibrio alginolyticus* from other literature.

Diagnosis	Characteristics of patients	Place	Epidemiology	References
Otorrhea	8-, 9-, and 47-year-old male patients	Hawaii, United States	Swimming in seawater	Pien et al., 1977
Otitis media	47-year-old man	Black-Sea	Contact with seawater	Ciufecu et al., 1979
Chronic otitis media	22-year-old man	Atlantic Ocean	Swimming in seawater	Hasyn et al., 1987
Acute otitis externa	2 patients (not detailed)	Mediterranean	Swimming in seawater	Dronda et al., 1991
Otitis externa	4 patients (not detailed)	Not mentioned	Contact with seawater	García-Martos et al., 1993
Otitis	6-year-old boy with bilateral tympanostomies	Gulf coast	Swimming in seawater	Levine et al., 1993
Suppurative otitis externa	21-year-old woman	Thermaikos Gulf, northern Greece	Diving in seawater	Tsakris et al., 1995
Acute suppurative otitis externa	51-year-old man	Lake Erie, United States	Water skiing	Mukherji et al., 2000
Acute otitis media (left)	4-year-old boy	Florida, United States	Swimming in seawater	Feingold and Kumar, 2004
Otitis media	57-year-old man	Mediterranean	Swimming in seawater	Ardiç and Ozyurt, 2004
Otitis	11-year-old boy	Oosterschelde, North Sea, Netherlands	Swimming in seawater	Schets et al., 2006
Chronic myringitis	47-year-old woman	Turkey	Bathing in seawater	Citil et al., 2015

In China, *V. alginolyticus*-associated gastro-intestinal diseases and wound infections are commonly reported. However, no reports of *Vibrio*-associated ear infections in China or any other Asian country have been published. Otolaryngologists may have insufficient awareness of how to properly collect microbiological specimens, in addition to a superficial understanding of rare pathogens that can cause ear infections. Ear swabs are not routinely performed and are not typically the specimen of choice for the diagnosis of otitis externa because swabs can also collect extraneous commensal microbes; therefore, swabs are often only used for recurrent episodes of severe otitis externa, chronic otitis, or in immunosuppressed individuals and patients who do not respond to initial therapy (Rosenfeld et al., 2014). If normal flora is recovered from ear samples alongside pathogenic microbes, *Vibrio* colonies may be mistaken for another clinically insignificant isolate.

In this case, three major factors contributed to the prolonged and chronic ear infection of the patient. First, the coast of the Yellow Sea in Asia and the Mediterranean Sea in Europe are at very similar latitudes and likely feature similar marine flora, increasing the likelihood that *Vibrio* spp. would be highly abundant in the temperate seawater with which the patient experienced contact during the summer season, increasing the probability of infection. Second, the ear-cleaning habit of the patient may have created epithelial lesions in the external auditory canal, facilitating the colonization and proliferation of this halophilic bacteria, resulting in infection. Finally, an indefinite diagnosis and insufficient treatment led to a prolonged course of infection. Many *Vibrio* spp. infections are self-limiting and do not require medical interventions beyond supportive care; however, the necessary treatment of these infections varies depending on the responsible pathogens and the observed clinical manifestations. Although overall experience managing chronic otitis externa caused by *V. alginolyticus* is limited, appropriate topical antibiotic agents are recommended to cure such otitis, according to a review of *Vibrio*-caused infections (Baker-Austin et al., 2018). Consistent with prior reports (Pien et al., 1977; Tsakris et al., 1995; Mukherji et al., 2000; Citil et al., 2015), the condition of our patient improved after antibiotic therapy.

In this study, we investigated the microbiological and otopathogenic characteristics of the *V. alginolyticus* strain, which was isolated from ear exudate specimens obtained from a patient with chronic otitis externa, to provide a basis for the future diagnosis of *V. alginolyticus*–associated infections. The identification of *V. alginolyticus* is accurate and reliable using a combination of MALDI-TOF MS, classical biochemical identification methods, *Vibrio*-selective media, and advanced molecular identification techniques, such as16S rRNA gene sequencing, facilitating the diagnosis of *V. alginolyticus*-associated infections. Furthermore, antimicrobial susceptibility testing revealed that the strain was resistant to ampicillin and sensitive to β-lactam, aminoglycosides, fluoroquinolones, and sulfonamide antibiotics. Overall, the microbiological identification and antibiotic-resistant phenotype of the *Vibrio* strain were consistent with the clinical diagnosis and prognosis of the patient.

Based on the history of bilateral otorrhea, the patient may have experienced infections in both ears due to *V. alginolyticus*, resulting in the mild loss of high-frequency hearing, which was severe in the right ear. No distinct evidence has been reported regarding the risks of sensorineural hearing loss due to *V. alginolyticus*-associated ear infections. To explore the potential otopathogenic effects of *V. alginolyticus*, we examined cell viability, apoptosis, and cell death in TM keratinocytes and HEI-OC1 cells treated with *V. alginolyticus*-conditioned medium *via* CCK-8 assay, wound-healing migration assay, flow cytometric analysis with Annexin V/PI double staining, and TUNEL staining. Our results indicated that the *V. alginolyticus* strain isolated from the patient could exert cytotoxic effects on TM keratinocytes and HEI-OC1 cells, inhibiting cell proliferation and migration and inducing cell apoptosis and death. To evaluate the ototoxicity of *V. alginolyticus* on HCs and SGNs, cell density and morphological integrity were analyzed after exposing cochlear organotypic explants to the bacteria supernatant. Our findings revealed the pre-dominant susceptibility of HCs and SGNs to ototoxic insults exerted by *V. alginolyticus* and the increased vulnerability of HCs and SGNs located at the basal cochlear region. Future investigations remain necessary to reveal the detailed mechanisms underlying *V. alginolyticus*-induced ototoxicity and to provide clinical solutions for effective prophylactic and therapeutic treatments of infection-associated hearing impairment.

In summary, our investigation highlights the challenges associated with the identification and characteristic analysis of the causative *Vibrio* strain found in this case, improving the understanding and awareness of clinicians and microbiologists when diagnosing *V. alginolyticus*-associated ear infections. Our report suggests that the clinicians, especially otolaryngologists, should be aware that *V. alginolyticus* may be a human pathogen, particularly among those patients who develop ear infections after participating in water-based activities, such as swimming, diving, fishing, or boating, or during any exposure to a marine environment or animals. Clinical microbiologists should also focus on suspected *Vibrio* colonies recovered from the ear samples of patients who have a history of seawater contact.

## Data Availability Statement

The original contributions presented in the study are included in the article/Supplementary Material, further inquiries can be directed to the corresponding authors.

## Ethics Statement

The studies involving human participants were reviewed and approved by the Ethics Committee of the Xijing Hospital, Fourth Military Medical University. The patients/participants provided their written informed consent to participate in this study. The animal study was reviewed and approved by the Animal Ethics Committee of Fourth Military Medical University. Written informed consent was obtained from the individual(s) for the publication of any potentially identifiable images or data included in this article.

## Author Contributions

KZ and FS conceived and designed the study. FS, K-YT, X-QL, and WL performed the experiments. KZ, K-YT, and X-YZ interpreted the data. KZ wrote the manuscript. FS and J-YL reviewed and edited the manuscript. All authors have read and approved the final manuscript.

## Conflict of Interest

The authors declare that the research was conducted in the absence of any commercial or financial relationships that could be construed as a potential conflict of interest.

## Publisher’s Note

All claims expressed in this article are solely those of the authors and do not necessarily represent those of their affiliated organizations, or those of the publisher, the editors and the reviewers. Any product that may be evaluated in this article, or claim that may be made by its manufacturer, is not guaranteed or endorsed by the publisher.

## Footnotes

1
http://blast.ncbi.nlm.nih.gov/Blast.cgi

## References

[B1] Ardiç N. Ozyurt M. (2004). Case report: otitis due to *Vibrio alginolyticus*. *Mikrobiyol. Bul.* 38 145–148. 15293914

[B2] Baker-Austin C. Oliver J. D. Alam M. Ali A. Waldor M. K. Qadri F. (2018). *Vibrio* spp. infections. *Nat. Rev. Dis. Primers* 4:8. 10.1038/s41572-018-0005-8 30002421

[B3] Barbarossa V. Kucisec-Tepes N. Aldova E. Matek D. Stipoljev F. (2002). Ilizarov technique in the treatment of chronic osteomyelitis caused by *Vibrio alginolyticus*. *Croat. Med. J.* 43 346–349. 12035144

[B4] Bhatkar D. Utpat K. Desai U. Joshi J. M. (2017). Bilateral tuberculous otitis media: an unique presentation. *Indian J. Tuberc.* 64 334–336. 10.1016/j.ijtb.2016.10.005 28941860

[B5] Bultmann C. A. Steiß J.-O. Langner C. Benkert B. Havener M. Küsters U. (2016). Complicated sea urchin-induced wound infection caused by *Vibrio alginolyticus* and *Staphylococcus lugdunensis* in a 14-year-old boy. *JMM Case Rep.* 3:e005074. 10.1099/jmmcr.0.005074 28348795 PMC5343123

[B6] Chen P.-J. Tseng C.-C. Chan H.-T. Chao C.-M. (2012). Acute Otitis due to *Vibrio fluvialis* after Swimming. *Case Rep. Emerg. Med.* 2012:838904. 10.1155/2012/838904 23326727 PMC3542905

[B7] Citil B. E. Derin S. Sankur F. Sahan M. Citil M. U. (2015). *Vibrio alginolyticus* associated chronic Myringitis acquired in mediterranean waters of Turkey. *Case Rep. Infect. Dis.* 2015:187212. 10.1155/2015/187212 26605095 PMC4641939

[B8] Ciufecu C. Năcescu N. Florescu D. (1979). Middle ear infection due to *Vibrio alginolyticus*. Bacteriological characterization. *Acta Microbiol. Acad. Sci. Hung.* 26 95–98. 484269

[B9] Ding D. He J. Allman B. L. Yu D. Jiang H. Seigel G. M. (2011). Cisplatin ototoxicity in rat cochlear organotypic cultures. *Hear. Res.* 282 196–203. 10.1016/j.heares.2011.08.002 21854840 PMC3230738

[B10] Dronda F. Cantón R. Selma J. L. García-Ramos F. Martínez-Ferrer M. (1991). Vibrio alginolyticus and swimmer’s otitis externa. 2 cases and review of the literature. *Enferm. Infecc. Microbiol. Clin*. 9, 630–633. 1822155

[B11] Feingold M. H. Kumar M. L. (2004). Otitis media associated with *Vibrio alginolyticus* in a child with pressure-equalizing tubes. *Pediatr. Infect. Dis. J.* 23 475–476. 10.1097/01.inf.0000126592.19378.30 15131479

[B12] García-Martos P. Benjumeda M. Delgado D. (1993). Otitis externa caused by *Vibrio alginolyticus*: description of 4 cases. *Acta Otorrinolaringol. Esp.* 44 55–57. 8471288

[B13] Gomez J. M. Fajardo R. Patiño J. F. Arias C. A. (2003). Necrotizing fasciitis due to *Vibrio alginolyticus* in an immunocompetent patient. *J. Clin. Microbiol.* 41 3427–3429. 10.1128/JCM.41.7.3427-3429.2003 12843111 PMC165324

[B14] Hasyn J. J. Mauer T. P. Warner R. Von Hake C. (1987). Isolation of *Vibrio alginolyticus* from a patient with chronic otitis media: report of case and review of biochemical activity. *J. Am. Osteopath. Assoc.* 87 560–562. 3667356

[B15] Jung J. Yoo J. E. Choe Y. H. Park S. C. Lee H. J. Lee H. J. (2019). Cleaved Cochlin Sequesters *Pseudomonas aeruginosa* and activates innate immunity in the inner Ear. *Cell Host Microbe* 25 513–525.e516. 10.1016/j.chom.2019.02.001 30905438

[B16] Kechker P. Senderovich Y. Ken-Dror S. Laviad-Shitrit S. Arakawa E. Halpern M. (2017). Otitis media caused by *V. cholerae* O100: a case report and review of the literature. *Front. Microbiol.* 8:1619. 10.3389/fmicb.2017.01619 28894440 PMC5581382

[B17] Lessner A. M. Webb R. M. Rabin B. (1985). *Vibrio alginolyticus* conjunctivitis: first reported case. *Archiv. Ophthalmol.* 103 229–230. 10.1001/archopht.1985.01050020081026 3977694

[B18] Levine W. C. Griffin P. M. Group G. C. V. W. (1993). *Vibrio* infections on the gulf coast: results of first year of regional surveillance. *J. Infect. Dis.* 167 479–483. 10.1093/infdis/167.2.479 8421186

[B19] Li X. C. Xiang Z. Y. Xu X. M. Yan W. H. Ma J. M. (2009). Endophthalmitis caused by *Vibrio alginolyticus*. *J. Clin. Microbiol.* 47 3379–3381. 10.1128/JCM.00722-09 19710275 PMC2756916

[B20] Liu X.-F. Zhang H. Liu X. Gong Y. Chen Y. Cao Y. (2014). Pathogenic analysis of *Vibrio alginolyticus* infection in a mouse model. *Folia Microbiol.* 59 167–171. 10.1007/s12223-013-0279-x 24065565

[B21] Mittal R. Lisi C. V. Gerring R. Mittal J. Mathee K. Narasimhan G. (2015). Current concepts in the pathogenesis and treatment of chronic suppurative otitis media. *J. Med. Microbiol.* 64 1103–1116. 10.1099/jmm.0.000155 26248613 PMC4835974

[B22] Mukherji A. Schroeder S. Deyling C. Procop G. W. (2000). An unusual source of *Vibrio alginolyticus*–associated otitis: prolonged colonization or freshwater exposure? *Archiv. Otolaryngol. Head Neck Surg.* 126 790–791. 10.1001/archotol.126.6.790 10864119

[B23] Nanda A. Zeki D. Parperis K. (2016). Chronic suppurative otitis media complicated with mastoiditis: an unusual presentation of tuberculosis. *Am. J. Med. Sci.* 352:544. 10.1016/j.amjms.2016.02.026 27865308

[B24] Neeff M. Biswas K. Hoggard M. Taylor M. W. Douglas R. Munson E. (2016). Molecular microbiological profile of chronic suppurative otitis media. *J. Clin. Microbiol.* 54 2538–2546. 10.1128/JCM.01068-16 27487953 PMC5035421

[B25] Opal S. M. Saxon J. R. (1986). Intracranial infection by *Vibrio alginolyticus* following injury in salt water. *J. Clin. Microbiol.* 23 373–374. 10.1128/jcm.23.2.373-374.1986 3700619 PMC268645

[B26] Orden B. Franco A. (1993). Isolation of *Vibrio alginolyticus* in 2 cases of otitis externa. *Enferm. Infecc. Microbiol. Clin.* 11 170–171. 8499526

[B27] Pien F. Lee K. Higa H. (1977). *Vibrio alginolyticus* infections in Hawaii. *J. Clin. Microbiol.* 5 670–672. 10.1128/jcm.5.6.670-672.1977 886005 PMC274679

[B28] Prakash Krishnan Muthaiah V. Ding D. Salvi R. Roth J. A. (2017). Carbaryl-induced ototoxicity in rat postnatal cochlear organotypic cultures. *Environ. Toxicol.* 32 956–969. 10.1002/tox.22296 27296064

[B29] Rosenfeld R. M. Schwartz S. R. Cannon C. R. Roland P. S. Simon G. R. Kumar K. A. (2014). Clinical practice guideline: acute otitis externa. *Otolaryngol. Head Neck Surg.* 150 S1–S24. 10.1177/0194599813517083 24491310

[B30] Ruiz C. C. Agraharkar M. (2003). Unusual marine pathogens causing Cellulitis and Bacteremia in Hemodialysis patients: report of two cases and review of the literature. *Hemodialysis Intern.* 7 356–359. 10.1046/j.1492-7535.2003.00063.x 19379389

[B31] Sato S. Miura T. Saitoh M. Tsukiashi S. Hongo T. Toba T. (2001). Identification of *Vibrio* vulnificus from patients with otitis media and septicemia by PCR method. *Kansenshogaku Zasshi* 75 307–313. 10.11150/kansenshogakuzasshi1970.75.307 11357321

[B32] Schets F. M. Van Den Berg H. H. Demeulmeester A. A. Van Dijk E. Rutjes S. A. Van Hooijdonk H. J. (2006). *Vibrio alginolyticus* infections in the Netherlands after swimming in the North Sea. *Euro Surveill.* 11:E061109.3. 10.2807/esw.11.45.03077-en 17213549

[B33] Sun F. Zhou K. Tian K.-Y. Wang J. Qiu J.-H. Zha D.-J. (2020). Atrial Natriuretic peptide improves neurite outgrowth from spiral ganglion neurons *in vitro* through a cGMP-Dependent Manner. *Neural Plast.* 2020:8831735. 10.1155/2020/8831735 33193754 PMC7643369

[B34] Sun F. Zhou K. Tian K.-Y. Zhang X.-Y. Liu W. Wang J. (2021). Atrial natriuretic peptide promotes neurite outgrowth and survival of cochlear spiral ganglion neurons *in vitro* through NPR-A/cGMP/PKG signaling. *Front. Cell Dev. Biol.* 9:681421. 10.3389/fcell.2021.681421 34268307 PMC8276373

[B35] Szmuilowicz J. Young R. (2019). Infections of the Ear. *Emerg. Med. Clin. N. Am.* 37 1–9. 10.1016/j.emc.2018.09.001 30454772

[B36] Taylor R. Mcdonald M. Russ G. Carson M. Lukaczynski E. (1981). *Vibrio alginolyticus* peritonitis associated with ambulatory peritoneal dialysis. *Br. Med. J.* 283 275–275. 10.1136/bmj.283.6286.275 6788288 PMC1506384

[B37] Tsakris A. Psifidis A. Douboyas J. (1995). Complicated suppurative otitis media in a Greek diver due to a marine halophilic *Vibrio* sp. *J. Laryngol. Otol.* 109 1082–1084. 10.1017/s0022215100132086 8551126

[B38] Uh Y. Park J. S. Hwang G. Y. Jang I. H. Yoon K. J. Park H. C. (2001). *Vibrio alginolyticus* acute gastroenteritis: report of two cases. *Clin. Microbiol. Infect.* 7 104–106. 10.1046/j.1469-0691.2001.00207.x 11298156

[B39] Wipperman J. (2014). Otitis externa. *Prim. Care Clin. Off. Pract.* 41 1–9. 10.1016/j.pop.2013.10.001 24439876

[B40] Wong A. C. Y. Ryan A. F. (2015). Mechanisms of sensorineural cell damage, death and survival in the cochlea. *Front. Aging Neurosci.* 7:58. 10.3389/fnagi.2015.00058 25954196 PMC4404918

