# Supplementary material for: Characteristic and Otopathogenic Analysis of a Vibrio alginolyticus Strain Responsible for Chronic Otitis Externa in China
Source: Front Microbiol. 2021 Dec 17;12:750642. doi: 10.3389/fmicb.2021.750642 (PMC8718755; doi:10.3389/fmicb.2021.750642)
Supplement: Supplementary file 3 [file Data_Sheet_1.DOCX]

Supplementary Material

**Characteristic and otopathogenic analysis of a *Vibrio alginolyticus* strain responsible for chronic otitis externa emerged in China**

**Ke Zhou^1†^, Ke-yong Tian^2†^, Xin-qin Liu^3†^, Wei Liu^2^,** **Xin-yu Zhang^2^, Jia-yun Liu^1*^, Fei Sun ^2*^**

^1^ Department of Laboratory Medicine, Institute of Clinical Laboratory Medicine of PLA, Xijing Hospital, Fourth Military Medical University, Xi’an, Shaanxi 710032, China

^2^ Department of Otolaryngology-Head and Neck Surgery, Xijing Hospital, Fourth Military Medical University, Xi’an, Shaanxi 710032, China

^3^ Department of Occupational and Environmental Health, Ministry of Education Key Lab of Hazard Assessment and Control in Special Operational Environment and Shaanxi Key Laboratory of Free Radical Biology and Medicine, School of Public Health, Fourth Military Medical University, Xi’an, Shaanxi 710032, China

***Correspondence:**

Fei Sun

susannajoke@163.com

Jia-yun Liu

jiayun@fmmu.edu.cn

# Supplementary figures


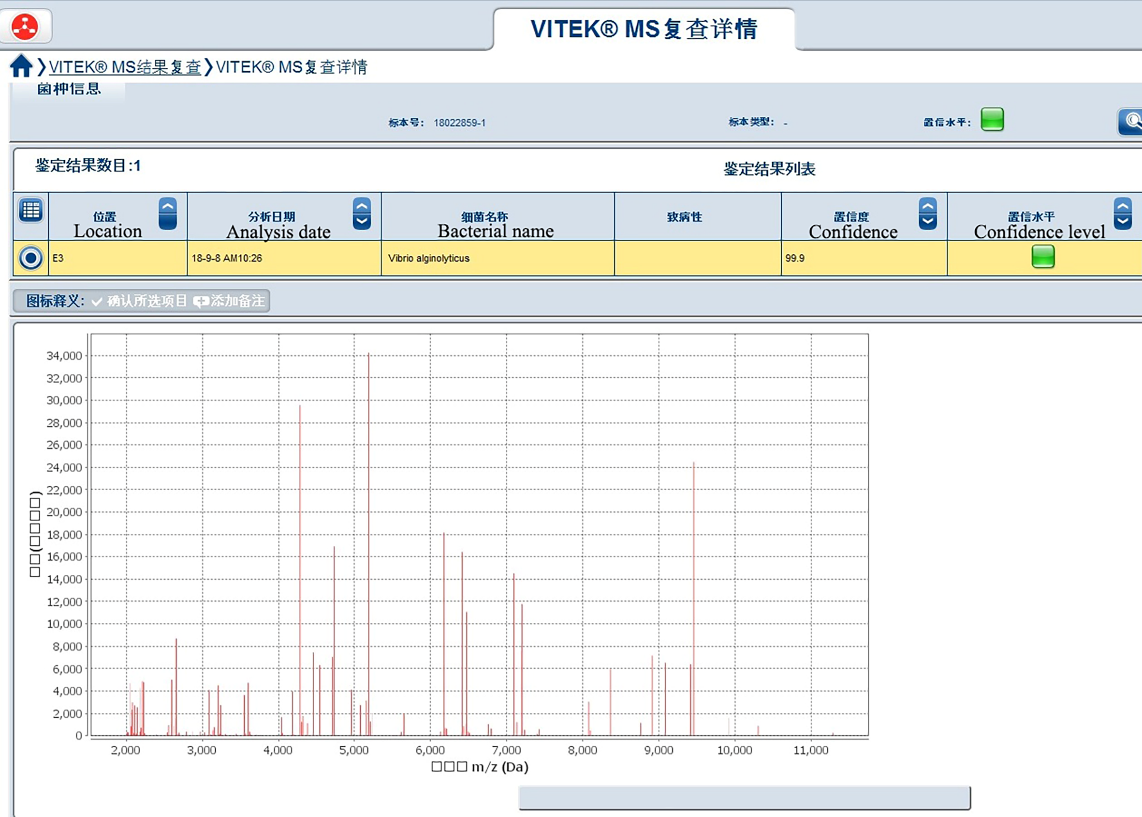


**Supplementary Figure 1** Result of bacterial identification by MALDI-TOF MS analysis and the obtained mass spectrometry spectra of the *V. alginolyticus* strain.

**Supplementary data 1. Identification results of 16S rRNA Gene Sequencing for the *Vibrio alginolyticus* strain.**

The sequence of the 16S rRNA gene was 100% identical (1407 bp over the entire 1407 bp fragment) with the *Vibrio alginolyticus* strain (FDAARGOS_114 chromosome 1, accession number CP014045.1).

CCCCGAAGGTTAAACTACCTACTTCTTTTGCAGCCCACTCCCATGGTGTGACGGGCGGTGTGTACAAGGCCCGGGAACGTATTCACCGTGGCATTCTGATCCACGATTACTAGCGATTCCGACTTCATGGAGTCGAGTTGCAGACTCCAATCCGGACTACGACGCACTTTTTGGGATTCGCTCACTCTCGCAAGTTGGCCGCCCTCTGTATGCGCCATTGTAGCACGTGTGTAGCCCTACTCGTAAGGGCCATGATGACTTGACGTCGTCCCCACCTTCCTCCGGTTTATCACCGGCAGTCTCCCTGGAGTTCCCGACATTACTCGCTGGCAAACAAGGATAAGGGTTGCGCTCGTTGCGGGACTTAACCCAACATTTCACAACACGAGCTGACGACAGCCATGCAGCACCTGTCTCAGAGTTCCCGAAGGCACCAATCCATCTCTGGAAAGTTCTCTGGATGTCAAGAGTAGGTAAGGTTCTTCGCGTTGCATCGAATTAAACCACATGCTCCACCGCTTGTGCGGGCCCCCGTCAATTCATTTGAGTTTTAATCTTGCGACCGTACTCCCCAGGCGGTCTACTTAACGCGTTAGCTCCGAAAGCCACGGCTCAAGGCCACAACCTCCAAGTAGACATCGTTTACGGCGTGGACTACCAGGGTATCTAATCCTGTTTGCTCCCCACGCTTTCGCATCTGAGTGTCAGTATCTGTCCAGGGGGCCGCCTTCGCCACCGGTATTCCTTCAGATCTCTACGCATTTCACCGCTACACCTGAAATTCTACCCCCCTCTACAGTACTCTAGTCTGCCAGTTTCAAATGCTATTCCGAGGTTGAGCCCCGGGCTTTCACATCTGACTTAACAAACCACCTGCATGCGCTTTACGCCCAGTAATTCCGATTAACGCTCGCACCCTCCGTATTACCGCGGCTGCTGGCACGGAGTTAGCCGGTGCTTCTTCTGTCGCTAACGTCAAATAATGCAGCTATTAACTACACTACCTTCCTCACGACTGAAAGTGCTTTACAACCCGAAGGCCTTCTTCACACACGCGGCATGGCTGCATCAGGCTTGCGCCCATTGTGCAATATTCCCCACTGCTGCCTCCCGTAGGAGTCTGGACCGTGTCTCAGTTCCAGTGTGGCTGATCATCCTCTCAGACCAGCTAGGGATCGTCGCCTTGGTGAGCCCTTACCTCACCAACTAGCTAATCCCACCTAGGCATATCCTGACGCGAGAGGCCCGAAGGTCCCCCTCTTTGGCCCGTAGGCATCATGCGGTATTAGCCATCGTTTCCAATGGTTATCCCCCACATCAGGGCAATTTCCTAGGCATTACTCACCCGTCCGCCGCTCGACGCCGTTATCGTTCCCCGAAGGTTCAGATAACTCGTTTCCGCTCGAC

| Identification results of 16S rRNA Gene Sequencing | | |
| --- | --- | --- |
| Sample ID | Identification results | Similarity of identification results（The top four most similar results） |
| 20180920  (18022859) | Vibrio alginolyticus strain； | Vibrio alginolyticus strain FDAARGOS_114 chromosome 1, complete sequence  Sequence ID: [CP014045.1](https://www.ncbi.nlm.nih.gov/nucleotide/CP014045.1?report=genbank&log$=nuclalign&blast_rank=1&RID=UP7D42RX015)Length: 3372141Number of Matches: 11  https://blast.ncbi.nlm.nih.gov/Blast.cgi#alnHdr_113015352 |
|  |  | Vibrio alginolyticus strain ATCC 33787 chromosome I, complete sequence  Sequence ID: [CP013484.1](https://www.ncbi.nlm.nih.gov/nucleotide/CP013484.1?report=genbank&log$=nuclalign&blast_rank=2&RID=UP7D42RX015)Length: 3362673Number of Matches: 10  https://blast.ncbi.nlm.nih.gov/Blast.cgi#alnHdr_113015352 |
|  |  | Vibrio sp. VibC-Oc-112 16S ribosomal RNA gene, partial sequence  Sequence ID: [KF577007.1](https://www.ncbi.nlm.nih.gov/nucleotide/KF577007.1?report=genbank&log$=nuclalign&blast_rank=3&RID=UP7D42RX015)Length: 1455Number of Matches: 1  https://blast.ncbi.nlm.nih.gov/Blast.cgi#alnHdr_113015352 |
|  |  | Vibrio sp. SMB7 16S ribosomal RNA gene, partial sequence  Sequence ID: [DQ868673.1](https://www.ncbi.nlm.nih.gov/nucleotide/DQ868673.1?report=genbank&log$=nuclalign&blast_rank=4&RID=UP7D42RX015)Length: 1459Number of Matches: 1  https://blast.ncbi.nlm.nih.gov/Blast.cgi#alnHdr_113015352 |

1，Range 1: 198869 to 200275[GenBank](https://www.ncbi.nlm.nih.gov/nucleotide/CP014045.1?report=genbank&log$=nuclalign&blast_rank=1&RID=UP7D42RX015&from=198869&to=200275)[Graphics](https://www.ncbi.nlm.nih.gov/nuccore/CP014045.1?report=graph&rid=UP7D42RX015%5bCP014045.1%5d&tracks=%5bkey:sequence_track,name:Sequence,display_name:Sequence,id:STD1,category:Sequence,annots:Sequence,ShowLabel:true%5d%5bkey:gene_model_track,CDSProductFeats:false%5d%5bkey:alignment_track,name:other%20alignments,annots:NG%20Alignments|Refseq%20Alignments|Gnomon%20Alignments|Unnamed,shown:false%5d&v=198799:200345&appname=ncbiblast&link_loc=fromHSP)Next MatchPrevious Match

| Alignment statistics for match #1 | | | | |
| --- | --- | --- | --- | --- |
| **Score** | **Expect** | **Identities** | **Gaps** | **Strand** |
| 2599 bits(1407) | 0.0 | 1407/1407(100%) | 0/1407(0%) | Plus/Minus |

Query 1 CCCCGAAGGTTAAACTACCTACTTCTTTTGCAGCCCACTCCCATGGTGTGACGGGCGGTG 60

||||||||||||||||||||||||||||||||||||||||||||||||||||||||||||

Sbjct 200275 CCCCGAAGGTTAAACTACCTACTTCTTTTGCAGCCCACTCCCATGGTGTGACGGGCGGTG 200216

Query 61 TGTACAAGGCCCGGGAACGTATTCACCGTGGCATTCTGATCCACGATTACTAGCGATTCC 120

||||||||||||||||||||||||||||||||||||||||||||||||||||||||||||

Sbjct 200215 TGTACAAGGCCCGGGAACGTATTCACCGTGGCATTCTGATCCACGATTACTAGCGATTCC 200156

Query 121 GACTTCATGGAGTCGAGTTGCAGACTCCAATCCGGACTACGACGCACTTTTTGGGATTCG 180

||||||||||||||||||||||||||||||||||||||||||||||||||||||||||||

Sbjct 200155 GACTTCATGGAGTCGAGTTGCAGACTCCAATCCGGACTACGACGCACTTTTTGGGATTCG 200096

Query 181 CTCACTCTCGCAAGTTGGCCGCCCTCTGTATGCGCCATTGTAGCACGTGTGTAGCCCTAC 240

||||||||||||||||||||||||||||||||||||||||||||||||||||||||||||

Sbjct 200095 CTCACTCTCGCAAGTTGGCCGCCCTCTGTATGCGCCATTGTAGCACGTGTGTAGCCCTAC 200036

Query 241 TCGTAAGGGCCATGATGACTTGACGTCGTCCCCACCTTCCTCCGGTTTATCACCGGCAGT 300

||||||||||||||||||||||||||||||||||||||||||||||||||||||||||||

Sbjct 200035 TCGTAAGGGCCATGATGACTTGACGTCGTCCCCACCTTCCTCCGGTTTATCACCGGCAGT 199976

Query 301 CTCCCTGGAGTTCCCGACATTACTCGCTGGCAAACAAGGATAAGGGTTGCGCTCGTTGCG 360

||||||||||||||||||||||||||||||||||||||||||||||||||||||||||||

Sbjct 199975 CTCCCTGGAGTTCCCGACATTACTCGCTGGCAAACAAGGATAAGGGTTGCGCTCGTTGCG 199916

Query 361 GGACTTAACCCAACATTTCACAACACGAGCTGACGACAGCCATGCAGCACCTGTCTCAGA 420

||||||||||||||||||||||||||||||||||||||||||||||||||||||||||||

Sbjct 199915 GGACTTAACCCAACATTTCACAACACGAGCTGACGACAGCCATGCAGCACCTGTCTCAGA 199856

Query 421 GTTCCCGAAGGCACCAATCCATCTCTGGAAAGTTCTCTGGATGTCAAGAGTAGGTAAGGT 480

||||||||||||||||||||||||||||||||||||||||||||||||||||||||||||

Sbjct 199855 GTTCCCGAAGGCACCAATCCATCTCTGGAAAGTTCTCTGGATGTCAAGAGTAGGTAAGGT 199796

Query 481 TCTTCGCGTTGCATCGAATTAAACCACATGCTCCACCGCTTGTGCGGGCCCCCGTCAATT 540

||||||||||||||||||||||||||||||||||||||||||||||||||||||||||||

Sbjct 199795 TCTTCGCGTTGCATCGAATTAAACCACATGCTCCACCGCTTGTGCGGGCCCCCGTCAATT 199736

Query 541 CATTTGAGTTTTAATCTTGCGACCGTACTCCCCAGGCGGTCTACTTAACGCGTTAGCTCC 600

||||||||||||||||||||||||||||||||||||||||||||||||||||||||||||

Sbjct 199735 CATTTGAGTTTTAATCTTGCGACCGTACTCCCCAGGCGGTCTACTTAACGCGTTAGCTCC 199676

Query 601 GAAAGCCACGGCTCAAGGCCACAACCTCCAAGTAGACATCGTTTACGGCGTGGACTACCA 660

||||||||||||||||||||||||||||||||||||||||||||||||||||||||||||

Sbjct 199675 GAAAGCCACGGCTCAAGGCCACAACCTCCAAGTAGACATCGTTTACGGCGTGGACTACCA 199616

Query 661 GGGTATCTAATCCTGTTTGCTCCCCACGCTTTCGCATCTGAGTGTCAGTATCTGTCCAGG 720

||||||||||||||||||||||||||||||||||||||||||||||||||||||||||||

Sbjct 199615 GGGTATCTAATCCTGTTTGCTCCCCACGCTTTCGCATCTGAGTGTCAGTATCTGTCCAGG 199556

Query 721 GGGCCGCCTTCGCCACCGGTATTCCTTCAGATCTCTACGCATTTCACCGCTACACCTGAA 780

||||||||||||||||||||||||||||||||||||||||||||||||||||||||||||

Sbjct 199555 GGGCCGCCTTCGCCACCGGTATTCCTTCAGATCTCTACGCATTTCACCGCTACACCTGAA 199496

Query 781 ATTCTACCCCCCTCTACAGTACTCTAGTCTGCCAGTTTCAAATGCTATTCCGAGGTTGAG 840

||||||||||||||||||||||||||||||||||||||||||||||||||||||||||||

Sbjct 199495 ATTCTACCCCCCTCTACAGTACTCTAGTCTGCCAGTTTCAAATGCTATTCCGAGGTTGAG 199436

Query 841 CCCCGGGCTTTCACATCTGACTTAACAAACCACCTGCATGCGCTTTACGCCCAGTAATTC 900

||||||||||||||||||||||||||||||||||||||||||||||||||||||||||||

Sbjct 199435 CCCCGGGCTTTCACATCTGACTTAACAAACCACCTGCATGCGCTTTACGCCCAGTAATTC 199376

Query 901 CGATTAACGCTCGCACCCTCCGTATTACCGCGGCTGCTGGCACGGAGTTAGCCGGTGCTT 960

||||||||||||||||||||||||||||||||||||||||||||||||||||||||||||

Sbjct 199375 CGATTAACGCTCGCACCCTCCGTATTACCGCGGCTGCTGGCACGGAGTTAGCCGGTGCTT 199316

Query 961 CTTCTGTCGCTAACGTCAAATAATGCAGCTATTAACTACACTACCTTCCTCACGACTGAA 1020

||||||||||||||||||||||||||||||||||||||||||||||||||||||||||||

Sbjct 199315 CTTCTGTCGCTAACGTCAAATAATGCAGCTATTAACTACACTACCTTCCTCACGACTGAA 199256

Query 1021 AGTGCTTTACAACCCGAAGGCCTTCTTCACACACGCGGCATGGCTGCATCAGGCTTGCGC 1080

||||||||||||||||||||||||||||||||||||||||||||||||||||||||||||

Sbjct 199255 AGTGCTTTACAACCCGAAGGCCTTCTTCACACACGCGGCATGGCTGCATCAGGCTTGCGC 199196

Query 1081 CCATTGTGCAATATTCCCCACTGCTGCCTCCCGTAGGAGTCTGGACCGTGTCTCAGTTCC 1140

||||||||||||||||||||||||||||||||||||||||||||||||||||||||||||

Sbjct 199195 CCATTGTGCAATATTCCCCACTGCTGCCTCCCGTAGGAGTCTGGACCGTGTCTCAGTTCC 199136

Query 1141 AGTGTGGCTGATCATCCTCTCAGACCAGCTAGGGATCGTCGCCTTGGTGAGCCCTTACCT 1200

||||||||||||||||||||||||||||||||||||||||||||||||||||||||||||

Sbjct 199135 AGTGTGGCTGATCATCCTCTCAGACCAGCTAGGGATCGTCGCCTTGGTGAGCCCTTACCT 199076

Query 1201 CACCAACTAGCTAATCCCACCTAGGCATATCCTGACGCGAGAGGCCCGAAGGTCCCCCTC 1260

||||||||||||||||||||||||||||||||||||||||||||||||||||||||||||

Sbjct 199075 CACCAACTAGCTAATCCCACCTAGGCATATCCTGACGCGAGAGGCCCGAAGGTCCCCCTC 199016

Query 1261 TTTGGCCCGTAGGCATCATGCGGTATTAGCCATCGTTTCCAATGGTTATCCCCCACATCA 1320

||||||||||||||||||||||||||||||||||||||||||||||||||||||||||||

Sbjct 199015 TTTGGCCCGTAGGCATCATGCGGTATTAGCCATCGTTTCCAATGGTTATCCCCCACATCA 198956

Query 1321 GGGCAATTTCCTAGGCATTACTCACCCGTCCGCCGCTCGACGCCGTTATCGTTCCCCGAA 1380

||||||||||||||||||||||||||||||||||||||||||||||||||||||||||||

Sbjct 198955 GGGCAATTTCCTAGGCATTACTCACCCGTCCGCCGCTCGACGCCGTTATCGTTCCCCGAA 198896

Query 1381 GGTTCAGATAACTCGTTTCCGCTCGAC 1407

|||||||||||||||||||||||||||

Sbjct 198895 GGTTCAGATAACTCGTTTCCGCTCGAC 198869

Range 2: 193593 to 194999[GenBank](https://www.ncbi.nlm.nih.gov/nucleotide/CP014045.1?report=genbank&log$=nuclalign&blast_rank=1&RID=UP7D42RX015&from=193593&to=194999)[Graphics](https://www.ncbi.nlm.nih.gov/nuccore/CP014045.1?report=graph&rid=UP7D42RX015%5bCP014045.1%5d&tracks=%5bkey:sequence_track,name:Sequence,display_name:Sequence,id:STD1,category:Sequence,annots:Sequence,ShowLabel:true%5d%5bkey:gene_model_track,CDSProductFeats:false%5d%5bkey:alignment_track,name:other%20alignments,annots:NG%20Alignments|Refseq%20Alignments|Gnomon%20Alignments|Unnamed,shown:false%5d&v=193523:195069&appname=ncbiblast&link_loc=fromHSP)Next MatchPrevious Match[First Match](https://blast.ncbi.nlm.nih.gov/Blast.cgi#hsp1345482739_1)

| Alignment statistics for match #2 | | | | |
| --- | --- | --- | --- | --- |
| **Score** | **Expect** | **Identities** | **Gaps** | **Strand** |
| 2593 bits(1404) | 0.0 | 1406/1407(99%) | 0/1407(0%) | Plus/Minus |

Query 1 CCCCGAAGGTTAAACTACCTACTTCTTTTGCAGCCCACTCCCATGGTGTGACGGGCGGTG 60

||||||||||||||||||||||||||||||||||||||||||||||||||||||||||||

Sbjct 194999 CCCCGAAGGTTAAACTACCTACTTCTTTTGCAGCCCACTCCCATGGTGTGACGGGCGGTG 194940

Query 61 TGTACAAGGCCCGGGAACGTATTCACCGTGGCATTCTGATCCACGATTACTAGCGATTCC 120

||||||||||||||||||||||||||||||||||||||||||||||||||||||||||||

Sbjct 194939 TGTACAAGGCCCGGGAACGTATTCACCGTGGCATTCTGATCCACGATTACTAGCGATTCC 194880

Query 121 GACTTCATGGAGTCGAGTTGCAGACTCCAATCCGGACTACGACGCACTTTTTGGGATTCG 180

||||||||||||||||||||||||||||||||||||||||||||||||||||||||||||

Sbjct 194879 GACTTCATGGAGTCGAGTTGCAGACTCCAATCCGGACTACGACGCACTTTTTGGGATTCG 194820

Query 181 CTCACTCTCGCAAGTTGGCCGCCCTCTGTATGCGCCATTGTAGCACGTGTGTAGCCCTAC 240

|||||| |||||||||||||||||||||||||||||||||||||||||||||||||||||

Sbjct 194819 CTCACTTTCGCAAGTTGGCCGCCCTCTGTATGCGCCATTGTAGCACGTGTGTAGCCCTAC 194760

Query 241 TCGTAAGGGCCATGATGACTTGACGTCGTCCCCACCTTCCTCCGGTTTATCACCGGCAGT 300

||||||||||||||||||||||||||||||||||||||||||||||||||||||||||||

Sbjct 194759 TCGTAAGGGCCATGATGACTTGACGTCGTCCCCACCTTCCTCCGGTTTATCACCGGCAGT 194700

Query 301 CTCCCTGGAGTTCCCGACATTACTCGCTGGCAAACAAGGATAAGGGTTGCGCTCGTTGCG 360

||||||||||||||||||||||||||||||||||||||||||||||||||||||||||||

Sbjct 194699 CTCCCTGGAGTTCCCGACATTACTCGCTGGCAAACAAGGATAAGGGTTGCGCTCGTTGCG 194640

Query 361 GGACTTAACCCAACATTTCACAACACGAGCTGACGACAGCCATGCAGCACCTGTCTCAGA 420

||||||||||||||||||||||||||||||||||||||||||||||||||||||||||||

Sbjct 194639 GGACTTAACCCAACATTTCACAACACGAGCTGACGACAGCCATGCAGCACCTGTCTCAGA 194580

Query 421 GTTCCCGAAGGCACCAATCCATCTCTGGAAAGTTCTCTGGATGTCAAGAGTAGGTAAGGT 480

||||||||||||||||||||||||||||||||||||||||||||||||||||||||||||

Sbjct 194579 GTTCCCGAAGGCACCAATCCATCTCTGGAAAGTTCTCTGGATGTCAAGAGTAGGTAAGGT 194520

Query 481 TCTTCGCGTTGCATCGAATTAAACCACATGCTCCACCGCTTGTGCGGGCCCCCGTCAATT 540

||||||||||||||||||||||||||||||||||||||||||||||||||||||||||||

Sbjct 194519 TCTTCGCGTTGCATCGAATTAAACCACATGCTCCACCGCTTGTGCGGGCCCCCGTCAATT 194460

Query 541 CATTTGAGTTTTAATCTTGCGACCGTACTCCCCAGGCGGTCTACTTAACGCGTTAGCTCC 600

||||||||||||||||||||||||||||||||||||||||||||||||||||||||||||

Sbjct 194459 CATTTGAGTTTTAATCTTGCGACCGTACTCCCCAGGCGGTCTACTTAACGCGTTAGCTCC 194400

Query 601 GAAAGCCACGGCTCAAGGCCACAACCTCCAAGTAGACATCGTTTACGGCGTGGACTACCA 660

||||||||||||||||||||||||||||||||||||||||||||||||||||||||||||

Sbjct 194399 GAAAGCCACGGCTCAAGGCCACAACCTCCAAGTAGACATCGTTTACGGCGTGGACTACCA 194340

Query 661 GGGTATCTAATCCTGTTTGCTCCCCACGCTTTCGCATCTGAGTGTCAGTATCTGTCCAGG 720

||||||||||||||||||||||||||||||||||||||||||||||||||||||||||||

Sbjct 194339 GGGTATCTAATCCTGTTTGCTCCCCACGCTTTCGCATCTGAGTGTCAGTATCTGTCCAGG 194280

Query 721 GGGCCGCCTTCGCCACCGGTATTCCTTCAGATCTCTACGCATTTCACCGCTACACCTGAA 780

||||||||||||||||||||||||||||||||||||||||||||||||||||||||||||

Sbjct 194279 GGGCCGCCTTCGCCACCGGTATTCCTTCAGATCTCTACGCATTTCACCGCTACACCTGAA 194220

Query 781 ATTCTACCCCCCTCTACAGTACTCTAGTCTGCCAGTTTCAAATGCTATTCCGAGGTTGAG 840

||||||||||||||||||||||||||||||||||||||||||||||||||||||||||||

Sbjct 194219 ATTCTACCCCCCTCTACAGTACTCTAGTCTGCCAGTTTCAAATGCTATTCCGAGGTTGAG 194160

Query 841 CCCCGGGCTTTCACATCTGACTTAACAAACCACCTGCATGCGCTTTACGCCCAGTAATTC 900

||||||||||||||||||||||||||||||||||||||||||||||||||||||||||||

Sbjct 194159 CCCCGGGCTTTCACATCTGACTTAACAAACCACCTGCATGCGCTTTACGCCCAGTAATTC 194100

Query 901 CGATTAACGCTCGCACCCTCCGTATTACCGCGGCTGCTGGCACGGAGTTAGCCGGTGCTT 960

||||||||||||||||||||||||||||||||||||||||||||||||||||||||||||

Sbjct 194099 CGATTAACGCTCGCACCCTCCGTATTACCGCGGCTGCTGGCACGGAGTTAGCCGGTGCTT 194040

Query 961 CTTCTGTCGCTAACGTCAAATAATGCAGCTATTAACTACACTACCTTCCTCACGACTGAA 1020

||||||||||||||||||||||||||||||||||||||||||||||||||||||||||||

Sbjct 194039 CTTCTGTCGCTAACGTCAAATAATGCAGCTATTAACTACACTACCTTCCTCACGACTGAA 193980

Query 1021 AGTGCTTTACAACCCGAAGGCCTTCTTCACACACGCGGCATGGCTGCATCAGGCTTGCGC 1080

||||||||||||||||||||||||||||||||||||||||||||||||||||||||||||

Sbjct 193979 AGTGCTTTACAACCCGAAGGCCTTCTTCACACACGCGGCATGGCTGCATCAGGCTTGCGC 193920

Query 1081 CCATTGTGCAATATTCCCCACTGCTGCCTCCCGTAGGAGTCTGGACCGTGTCTCAGTTCC 1140

||||||||||||||||||||||||||||||||||||||||||||||||||||||||||||

Sbjct 193919 CCATTGTGCAATATTCCCCACTGCTGCCTCCCGTAGGAGTCTGGACCGTGTCTCAGTTCC 193860

Query 1141 AGTGTGGCTGATCATCCTCTCAGACCAGCTAGGGATCGTCGCCTTGGTGAGCCCTTACCT 1200

||||||||||||||||||||||||||||||||||||||||||||||||||||||||||||

Sbjct 193859 AGTGTGGCTGATCATCCTCTCAGACCAGCTAGGGATCGTCGCCTTGGTGAGCCCTTACCT 193800

Query 1201 CACCAACTAGCTAATCCCACCTAGGCATATCCTGACGCGAGAGGCCCGAAGGTCCCCCTC 1260

||||||||||||||||||||||||||||||||||||||||||||||||||||||||||||

Sbjct 193799 CACCAACTAGCTAATCCCACCTAGGCATATCCTGACGCGAGAGGCCCGAAGGTCCCCCTC 193740

Query 1261 TTTGGCCCGTAGGCATCATGCGGTATTAGCCATCGTTTCCAATGGTTATCCCCCACATCA 1320

||||||||||||||||||||||||||||||||||||||||||||||||||||||||||||

Sbjct 193739 TTTGGCCCGTAGGCATCATGCGGTATTAGCCATCGTTTCCAATGGTTATCCCCCACATCA 193680

Query 1321 GGGCAATTTCCTAGGCATTACTCACCCGTCCGCCGCTCGACGCCGTTATCGTTCCCCGAA 1380

||||||||||||||||||||||||||||||||||||||||||||||||||||||||||||

Sbjct 193679 GGGCAATTTCCTAGGCATTACTCACCCGTCCGCCGCTCGACGCCGTTATCGTTCCCCGAA 193620

Query 1381 GGTTCAGATAACTCGTTTCCGCTCGAC 1407

|||||||||||||||||||||||||||

Sbjct 193619 GGTTCAGATAACTCGTTTCCGCTCGAC 193593

# Supplementary videos

**Supplementary Video 1** A time-lapse video of wound-healing migration assay was performed by analyzing repair rates of wound gaps between health TM keratinocytes maintained in the control condition. Note that the healthy epidermal cells maintained in normal medium proliferated and migrated to close the wound gap created by the 2-well culture insert over 24 h.

**Supplementary Video 2** A time-lapse video of wound-healing migration assay for wound gaps between TM keratinocytes maintained in *Vibrio*- derived conditioned medium. Note that the repair rate for wound-healing of keratinocytes treated with *Vibrio*-supernatant was significantly reduced in comparison to the normal control.
